# Relationship between diagnosis of conus arteriosus malformation and genetic diagnosis results in fetal cardiac axis abnormalities by echocardiography during middle pregnancy

**DOI:** 10.3389/fcvm.2024.1377095

**Published:** 2024-05-20

**Authors:** Lin Yang, Yuting Cai, Huie Chen, Linfang Ke, Shufen Wu

**Affiliations:** ^1^ Department of Ultrasound, Zhangzhou Affiliated Hospital of Fujian Medical University, Zhangzhou, China; ^2^ Department of Obstetrics, Zhangzhou Affiliated Hospital of Fujian Medical University, Zhangzhou, China

**Keywords:** echocardiography, fetus, cardiac axis, conus arteriosus malformation, genetics, relationship

## Abstract

**Objective:**

To explore the clinical value of echocardiography in detecting fetal cardiac axis abnormalities during middle pregnancy for diagnosing conus arteriosus malformation, and to compare and analyze the genetic diagnosis results, in order to provide evidence for clinical diagnosis and intervention.

**Methods:**

Four hundred twenty-one fetuses with conus arteriosus malformation from January 2020 to October 2023 were included as the conus arteriosus malformation group, and 917 healthy fetuses (all single fetuses) matched at the same gestational age were selected as the healthy group.

**Results:**

There was no significant difference in gestational weeks between two groups (*P* > 0.05). The age of pregnant women in conus arteriosus malformation group was lower compared to healthy group (*P* < 0.05), and the fetal cardiac axis in conus arteriosus malformation group was significantly higher compared to healthy group (*P* < 0.05). Among the fetuses with conus arteriosus malformation, tetralogy of Fallot (TOF), transposition of the great arteries (TGA) and double outlet right ventricle (DORV) had the highest proportions, accounting for 38.00%, 18.29% and 17.58%, respectively. Among all types of conus arteriosus malformations, atresia pulmonary valve syndrome associated with TOF, persistent truncus arteriosus and DORV exhibited higher proportions of fetal cardiac axis abnormalities, at 75.00%, 36.84% and 27.03%, respectively, while TGA and interrupted aortic arch associated with B-type interruption had lower proportions of fetal cardiac axis abnormalities, at 2.60% and 4.55%, respectively. Genetic testing was conducted on 73 cases (17.34%) of fetuses with conus arteriosus malformation in this study. Among them, fetal cardiac axis abnormalities were considered positive for genetic results due to factors such as aneuploidy, copy number abnormalities, and single-gene pathogenicity. A total of 31 cases tested positive for genetic anomalies, with a positive rate of approximately 42.47%.

**Conclusion:**

In the middle pregnancy, the fetal cardiac axis in cases of conus arteriosus malformation was significantly higher than in normal fetuses. Moreover, there were variations in fetal cardiac axis among different types of conus arteriosus malformations, and these differences were notably associated with genetic diagnostic results.

## 1 Introduction

With the continuous development of medical technology, the diagnostic rate of fetal cardiovascular disease has gradually increased. Conus arteriosus malformations are a group of congenital heart defects involving the outflow tracts of the heart and great vessels, a complex type of congenital heart disease that is a leading cause of neonatal mortality, and is associated with the main ventricular and outflow tract regions of the heart. It usually refers to a portion of the right ventricular outflow tract that leads from the right ventricle to the pulmonary artery. Anomalies of the conus arteriosus are observed in various congenital heart diseases, such as tetralogy of Fallot (TOF), double outlet right ventricle (DORV), transposition of the great arteries (TGA), and pulmonary artery atresia with ventricular septal defect (PAVSD), and are often accompanied by outflow tract obstruction (1, 2). Cardiac imaging technology is widely used in the diagnosis and treatment of heart disease at present. In the clinical diagnosis of conus arteriosus malformation, options include echocardiography, CT angiography, cardiac MRI and cardiac catheterization. Among them, echocardiography has the advantages of simple operation, non-invasive, non-radiation and repeatable, and is currently the most commonly used imaging tool for the diagnosis of conus arteriosus malformation (3, 4). The cardiac axis refers to the direction of the heart within the chest. Some scholars believe that the fetal axis of the heart is closely related to many congenital heart diseases, especially the occurrence of conus arteriosus malformation. However, there are few studies on the relationship between the fetal axis of the heart and conus arteriosus malformation in China (5, 6). Cardiac axis abnormality refers to the change of the arrangement of the heart on the vertical axis, while conus arteriosus malformation refers to abnormalities in the development of the aorta and pulmonary artery, which may lead to the death of the fetus or require complex cardiac surgery after birth (7). Therefore, early diagnosis and intervention are of paramount importance. This study included 1,338 pregnant women who underwent echocardiography in our hospital from January 2020 to October 2023 as research objects, aiming to analyze the clinical value of echocardiography in detecting fetal cardiac axis abnormalities during middle pregnancy for diagnosing conus arteriosus malformation, and to compare it with the genetic diagnosis results. The results of this study may help to improve the diagnostic accuracy of fetal cardiac axis abnormalities and conus arteriosus malformation, and provide more information for families and doctors to carry out more accurate intervention and planning during pregnancy. In addition, this study also contributes to a better understanding of the genetic mechanisms underlying conus arteriosus malformation, offering new insights for the prevention and treatment of inherited cardiovascular diseases in the future.

## 2 Materials and methods

### 2.1 General information

1,338 pregnant women who underwent echocardiography examination in our hospital from January 2020 to October 2023 were included in the study. 421 fetuses with conus artery trunk malformation were selected as the conus artery trunk malformation group, excluding fetuses with conus artery trunk malformation combined with dextrocardia and extramediastinal malformations. 917 normal fetuses (all single fetuses) matched at the same gestational age were selected as the healthy group. The inclusion criteria were as follows: (1) Complete prenatal data, in the mid-pregnancy period; (2) singleton pregnancies only; (3) all participants underwent chromosomal testing. Exclusion criteria: (1) Presence of clear etiological factors such as intrauterine infection or teratogenic exposure; (2) existence of a family history of genetic diseases. The operation of this experiment was approved by the hospital's ethics committee.

### 2.2 Observation indicators

General data: gestational weeks and age of pregnant women were collected and compared between two groups.

Fetal echocardiography was performed in all patients according to sequential segmental approach. Echocardiographic tests (8): GE Voluson E8 Doppler ultrasound diagnostic instrument was used to perform echocardiography. The probe frequency was 2.0MHZ-3.5MHZ. Fetal cardiac axis measurements were compared between the two groups and among fetuses with different types of conus arteriosus malformations. An axis measurement above 65° was considered enlarged/deviated, while below 25° was considered reduced, both indicating abnormal diagnosis (9). Two measurements were taken, and the average value was calculated.

Conus arteriosus malformation included TOF, TGA, DORV, pulmonary atresia with ventricular septal defect (PA-VSD), persistent truncus arteriosus (PTA), aortic pulmonary septal defect (APSD), interrupted aortic arch associated with B-type interruption (IAA-B), atresia pulmonary valve syndrome associated with TOF (APVS-TOF), etc.

Genetic testing: Low depth whole genome and whole exome sequencing technology were used for diagnosis by amniotic fluid puncture (10). Low depth whole genome sequencing refers to the use of high throughput sequencing technology to conduct low depth whole genome sequencing of sample DNA, compare the sequencing results with the human reference genome base sequence, and detect the presence of CNVs in the tested sample through biological information analysis (11).Among them, fetal cardiac axis abnormalities were considered positive for genetic results due to factors such as aneuploidy, copy number abnormalities, and single-gene pathogenicity.

Repeat testing between and within observers: 30 cases were selected by two deputy senior physicians and above, each case was measured independently by two investigators, and again by a physician two weeks later, followed by a conformance test.

### 2.3 Statistical analysis

SPSS20.0 software was used to analyze the experimental data. The measurement data of maternal age, fetal cardiac axis and gestational weeks were expressed by $\left(\bar{x}\pm s\right)$, and *t*-test was adopted. The enumeration data of fetal cardiac axis abnormality were represented by (%), using chi-^2^square test. The relationship between fetal cardiac axis and genetic test results was analyzed using multivariate Logistic regression. The value of fetal cardiac axis in predicting the coexistence of conus arteriosus malformation and genetic abnormalities was assessed using the receiver operating characteristic (ROC) curve. The intraclass correlation coefficient (ICC) was utilized for consistency testing of measurements among different observers, with an ICC value of ≥0.75 indicating good consistency between the two. Statistical results with *P < 0.*05 were considered to be statistically significant.

## 3 Results

### 3.1 Comparison of clinical data and fetal cardiac axis

**Figure 1:**
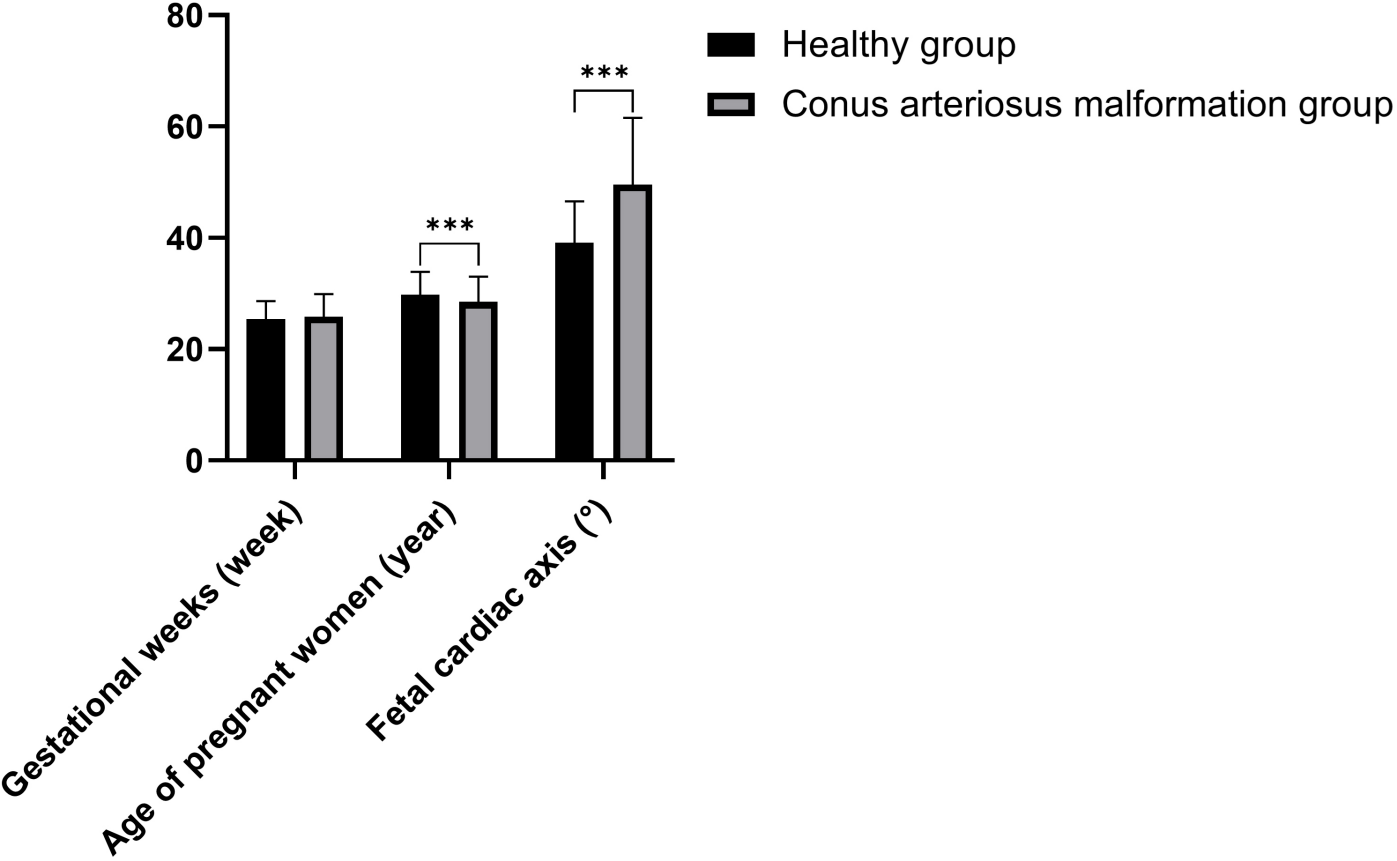
Comparison of clinical data and fetal cardiac axis (***P < 0.001).

The age of pregnant women in conus arteriosus malformation group was lower compared to healthy group (*P* < 0.05); the fetal cardiac axis in conus arteriosus malformation group was significantly higher in contrast to healthy group (*P* < 0.05). See Table 1 and Figure 1.

**Table 1:** Comparison of clinical data and fetal cardiac axis $\left(\bar{x}\pm s\right)$.

Group	Gestational weeks (week)	Age of pregnant women (year)	Fetal cardiac axis (°)
Healthy group (*n* = 917)	25.46 ± 3.15	29.76 ± 4.13	39.12 ± 7.48
Conus arteriosus malformation group (*n* = 421)	25.81 ± 4.06	28.52 ± 4.50	49.56 ± 12.00
*t*	1.717	4.956	19.392
*P*	0.086	<0.001	<0.001

### 3.2 Comparison of fetal cardiac axis abnormalities in different types of conus arteriosus malformations

**Figure 2:**
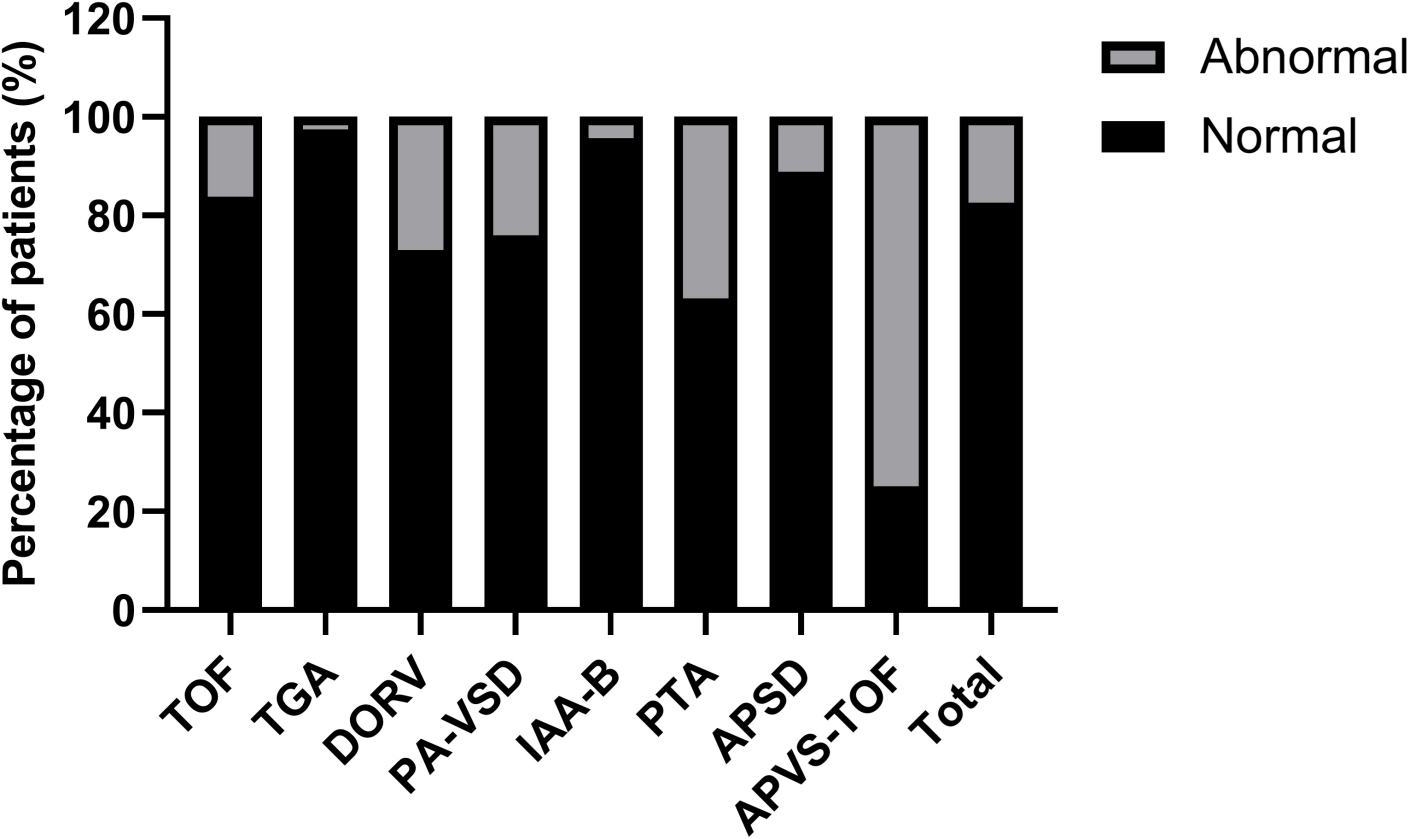
Comparison of fetal cardiac axis abnormalities in different types of conus arteriosus malformations.

Among fetuses with conus arteriosus malformation, TOF fetuses account for 38.00%, TGA fetuses account for 18.29%, and DORV fetuses account for 17.58%. Among all types of conus arteriosus malformation, APVS-TOF, PTA, and DORV fetuses have a higher proportion of abnormal cardiac axis, accounting for 75.00%, 36.84%, and 27.03%, respectively. TGA and IAA-B fetuses have a lower proportion of abnormal cardiac axis, accounting for 2.60% and 4.55%, respectively. See Table 2 and Figure 2.

**Table 2:** Comparison of fetal cardiac axis abnormalities in different types of conus arteriosus malformations (*n*, %).

Type	Total cases	Fetal cardiac axis	
		Normal	Abnormal
TOF	161	135 (83.85)	26 (16.15)
TGA	77	75 (97.40)	2 (2.60)
DORV	74	54 (72.97)	20 (27.03)
PA-VSD	54	41 (75.93)	13 (24.07)
IAA-B	23	22 (95.65)	1 (4.35)
PTA	19	12 (63.16)	7 (36.84)
APSD	9	8 (88.89)	1 (11.11)
APVS-TOF	4	1 (25.00)	3 (75.00)
Total	421	348 (82.66)	73 (17.34)

TOF, tetralogy of Fallot; TGA, transposition of the great arteries; DORV, double outlet right ventricle; PA-VSD, pulmonary atresia with ventricular septal defect; IAA-B, interrupted aortic arch associated with B-type interruption; PTA, persistent truncus arteriosus; APSD, aortic pulmonary septal defect; APVS-TOF, atresia pulmonary valve syndrome associated with TOF.

### 3.3 Comparison of cardiac axis between fetuses with different subtypes of conus arteriosus malformations and healthy fetuses

Compared with healthy group, the cardiac axis levels of TOF, DORV, PA-VSD, PTA, APSD and APVS-TOF types of conus arteriosus malformations were significantly higher (*P* < 0*.*05). See Table 3.

**Table 3:** Comparison of cardiac axis between fetuses with different subtypes of conus arteriosus malformations and healthy fetuses $\left(\bar{x}\pm s\right)$.

Group	Case	Fetal cardiac axis (°)	*t*	*P*
TOF	160	50.60 ± 13.28	15.602	<0.001
TGA	77	39.15 ± 8.42	0.034	0.973
DORV	74	52.19 ± 15.48	12.972	<0.001
PA-VSD	54	57.16 ± 13.05	16.334	<0.001
IAA-B	23	38.40 ± 6.19	0.458	0.647
PTA	19	58.02 ± 7.03	10.914	<0.001
APSD	9	58.01 ± 12.19	7.486	<0.001
APVS-TOF	4	57.16 ± 24.05	4.741	<0.001
Healthy group	917	39.12 ± 7.48	–	–

*t* is for comparison with healthy group, *P* < 0.05 is statistically significant.

TOF, tetralogy of Fallot; TGA, transposition of the great arteries; DORV, double outlet right ventricle; PA-VSD, pulmonary atresia with ventricular septal defect; IAA-B, interrupted aortic arch associated with B-type interruption; PTA, persistent truncus arteriosus; APSD, aortic pulmonary septal defect; APVS-TOF, atresia pulmonary valve syndrome associated with TOF.

### 3.4 Multivariate analysis of genetic abnormalities affecting fetal conus arteriosus malformation

**Figure 3:**
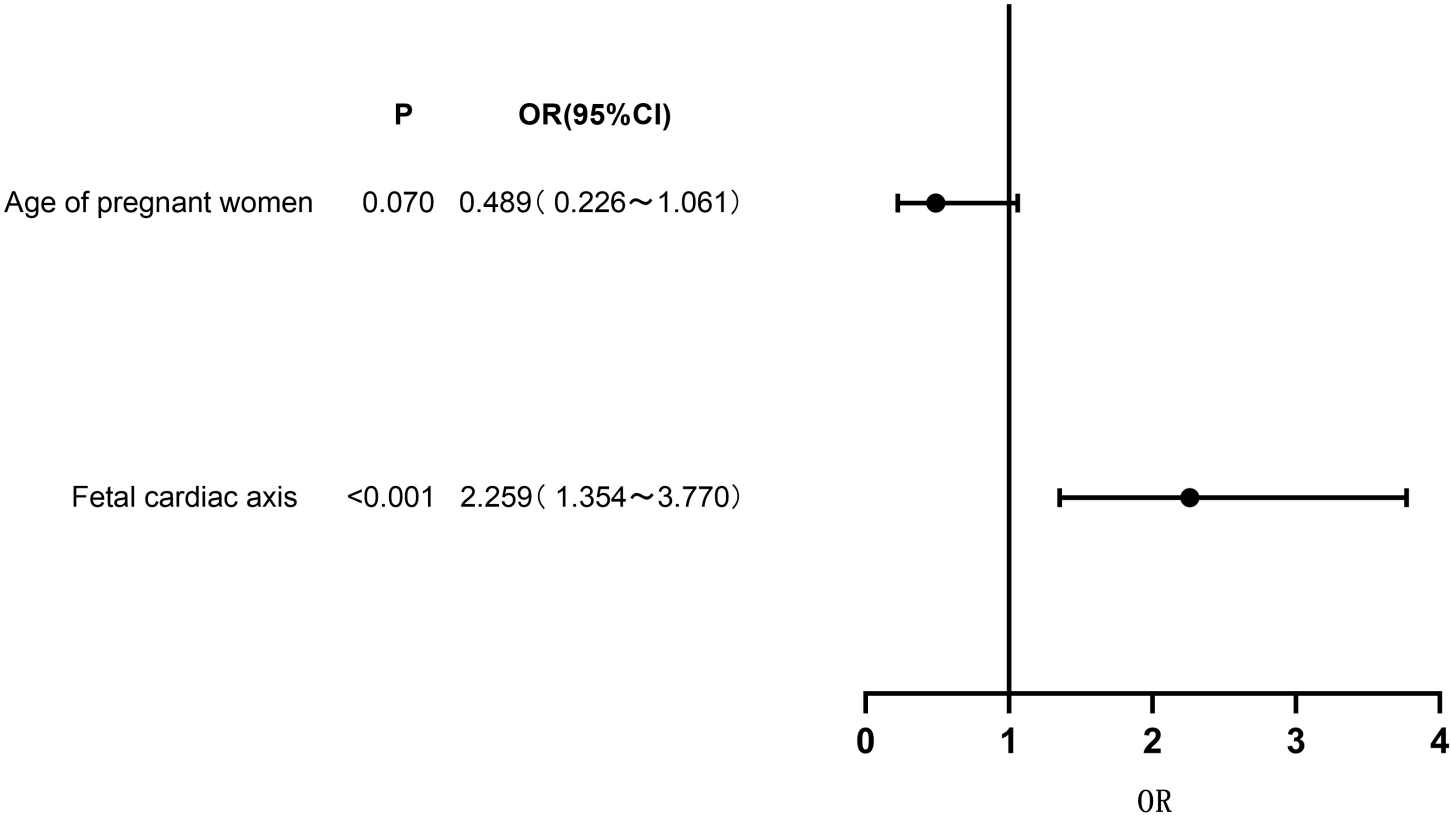
Forest plot of clinical characteristics based on multivariate logistic regression analysis.

Genetic testing was conducted on 73 cases (17.34%) of fetuses with conus arteriosus malformation in this study. A total of 31 cases tested positive for genetic anomalies, with a positive rate of approximately 42.47%. The results of multivariate Logistic regression analysis indicated a significant correlation between fetal cardiac axis and genetic abnormalities associated with conus arteriosus malformation (*P < *0*.*05). See Table 4 and Figure 3.

**Table 4:** Multivariate analysis of genetic abnormalities affecting fetal conus arteriosus malformation.

Index	β	SE	Wald *χ*^2^	*P*	OR	95% CI
Age of pregnant women	−0.715	0.395	3.277	0.070	0.489	0.226–1.061
Fetal cardiac axis	0.815	0.261	9.751	<0.001	2.259	1.354–3.770

### 3.5 Analysis of fetal consistency test for conus arteriosus malformation

**Figure 4:**
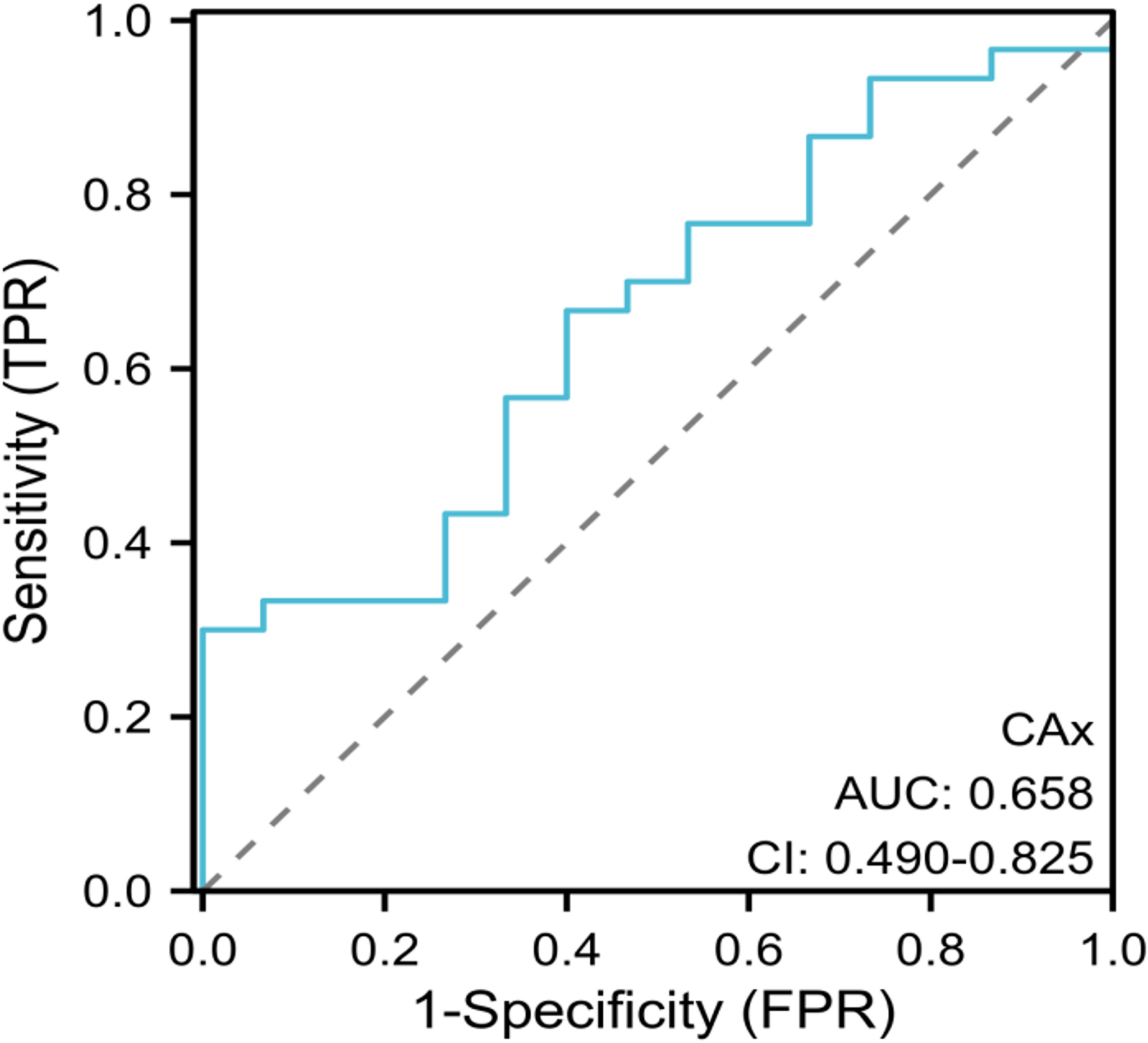
ROC curve analysis. ROC, receiver operating characteristic; AUC, area under the curve;CI, 95% confidence interval.

The established ROC curve revealed that the area under the curve (AUC) for fetal cardiac axis in predicting the the coexistence of conus arteriosus malformation and genetic abnormalities was 0.658 (95%CI: 0.490–0.825). When the cutoff value was set at 59.4°, the sensitivity was 62.15% and the specificity was 60.08%, and the Youden index was 0.223. The ICC consistency test results demonstrated high consistency in the measurement of fetal cardiac axis between observers and within observers. See Figure 4.

## 4 Discussion

Conus arteriosus refers to a conical structure from the right ventricle to the pulmonary trunk, and conus arteriosus malformation is a common type of congenital heart disease (2, 12). As a complex congenital heart disease, it includs the development of the conus arteriosus and the relative position of the major arteries two types of abnormalities. This condition poses a serious threat to the life safety of newborns and is one of the main causes of neonatal death (13, 14). Echocardiography has good spatial and temporal resolution, and can clearly display the various structures of the fetal heart, providing a strong imaging basis for clinical diagnosis. Some studies have found that in some cases of abnormal cardiac axis detected by echocardiography, the genetic diagnosis result of some cases is negative, suggesting that there may be false positive phenomenon in the diagnosis of abnormal cardiac axis by echocardiography (15, 16). In addition, some studies have found that the genetic diagnosis results of some cases of cardiac axis abnormalities are not consistent with the results of echocardiography, suggesting that the genetic diagnosis may not fully explain the cardiac axis abnormalities detected by echocardiography in some cases (17, 18). Therefore, exploring the relationship between echocardiography and genetic diagnosis results is of great significance for optimizing diagnostic strategies for fetal cardiac axis abnormalities.

Fetal cardiac axis is one of the components examined in echocardiography.The measurement of the fetal cardiac axis is relatively simple and easily obtainable, and it is a non-invasive and radiation-free procedure that allows for repeated operations (17, 19). Through the real-time dynamic observation of the fetal heart, echocardiography can clearly show the structure and function of the fetal heart, providing an important basis for clinical diagnosis. Conus arteriosus malformation is a complex heart disease caused by abnormal development of cardiac neural crest cells, resulting in abnormal development of conus arteriosus, excessive rotation of fetal ventricular ring and insufficient development of thymus can lead to fetal cardiac axis abnormalities (20, 21). Moreover, the structural normality of conus arteriosus malformation in the four-chamber heart section often escapes early attention from operators. However, echocardiography allows for accurate measurement of the fetal cardiac axis when displaying the standard four-chamber heart section, aiding in early recognition and further diagnostic screening (22). In this study, conus arteriosus malformation group had significantly elevated fetal cardiac axis compared with healthy group, indicating that abnormal fetal cardiac axis level is common in fetuses with conus arteriosus malformation, which was similar to the results of Oşvar FN et al. (23), who suggested that fetal cardiac axis had a high diagnostic value for detecting congenital heart anomalies such as nuchal translucency, tricuspid regurgitation, and venous wave inversion A-wave. A fetal cardiac axis measurement greater than 57° suggested a higher risk of conus arteriosus malformations or major vascular abnormalities. This study also showed that among all types of conus arteriosus malformations, APVS-TOF, PTA and DORV accounted for a relatively high proportion of fetal cardiac axis abnormalities, accounting for 75.00%, 36.84% and 27.03%, respectively, while TGA and IAA-B had a relatively low proportion of fetal cardiac axis abnormalities, accounting for 2.60% and 4.55%, respectively. The fetal cardiac axis levels of TOF, DORV, PA-VSD, PTA, APSD and APVS-TOF types of conus arteriosus malformations were significantly higher in contrast to healthy group. It was evident that fetal cardiac axis exhibited abnormal variations in various types of conus arteriosus malformations. Elevated expression of fetal cardiac axis may encompass multiple types of conus arteriosus malformations, aiding in early screening and diagnosis. This held significant importance for early intervention and guidance of maternal-fetal outcomes. In addition, it also indicated that echocardiography had important application value in the diagnosis of conus arteriosus malformation. Through detailed examination of the fetal heart, it could be found that the characteristic manifestations of conus arteriosus malformation, such as abnormal heart size and shape, spindle deviation, and structural abnormalities of cardiovascular valves and vascular rings. Echocardiography allows real-time observation of dynamic changes in the heart, providing crucial information for clinically assessing the severity of the condition, selecting appropriate treatment strategies, and predicting prognosis. The wide application of echocardiography has significantly improved the diagnostic accuracy of conus arteriosus malformation, which is conducive to reducing the mortality and disability rate of the fetuses.

Current study has found that the incidence of conus arteriosus malformation is closely related to genetic abnormalities. In the fetal heart development stage, TBX1 and DGCR6 genes are located in the deletion stage of chromosome 22, among which TBX1 is a transcription factor expressed in the germ layer of the pharynx. Fetal conus arteriosus malformation is closely related to chromosome 22q11.2 microdeletion (24). It has also been found that fetal cardiac axis abnormality is significantly correlated with fetal aneuploidy of congenital heart disease (25). The results of multivariate Logistic regression analysis in this study showed that fetal cardiac axis and conus arteriosus malformation were associated with genetic abnormalities. It was suggested that genetic changes such as chromosomal abnormalities and gene mutations may be the key factors leading to conus arteriosus malformation. Therefore, in the process of clinical diagnosis and intervention, genetic diagnosis is of great significance, helping to reveal the pathogenesis of conus arteriosus malformation, and providing scientific basis for the prevention and screening of familial cases. The established ROC curve showed that AUC for fetal cardiac axis in predicting the the coexistence of conus arteriosus malformation and genetic abnormalities was 0.658 (95%CI: 0.490–0.825). The results indicated that fetal cardiac axis abnormalities were closely related to genetic abnormalities, but the AUC was 0.658, indicating low diagnostic efficiency. Therefore, relevant indicators could be further searched for for screening and prediction of genetic abnormalities.

In summary, the fetal cardiac axis in cases of conus arteriosus malformation was significantly higher than in normal fetuses in the middle pregnancy. Moreover, there were variations in fetal cardiac axis among different types of conus arteriosus malformations, and these differences were notably associated with genetic diagnostic results.Future research could expand on the current study in the following aspects: (1) Increase the sample size to further verify the relationship between fetal cardiac axis abnormalities detected by echocardiography and conus arteriosus malformations; (2) conduct in-depth subgroup analyses of echocardiography results to explore the association between different types of fetal cardiac axis abnormalities and conus arteriosus malformations; (3) integrate additional genetic data to investigate the consistency between echocardiography results and genetic diagnosis results, along with exploring the underlying mechanisms; (4) conduct multi-center studies to enhance the generalizability and reliability of research findings. Through these endeavors, we aim to provide stronger evidence for the screening and diagnosis of fetal cardiac axis abnormalities during the mid-pregnancy period in clinical practice.

## Data Availability

The data presented in the study are deposited in the SRA database (https://www.ncbi.nlm.nih.gov/sra), accession number: SRA296708.
